# Supplementary material for: Boosting oxygen evolution of single-atomic ruthenium through electronic coupling with cobalt-iron layered double hydroxides
Source: Nat Commun. 2019 Apr 12;10:1711. doi: 10.1038/s41467-019-09666-0 (PMC6461613; doi:10.1038/s41467-019-09666-0)
Supplement: Supplementary file 1 — Supplementary Information [file 41467_2019_9666_MOESM1_ESM.pdf]

Supplementary Information

Boosting Oxygen Evolution of Single-Atomic Ruthenium through Electronic Coupling with Cobalt-Iron Layered Double Hydroxides

Li et al.

Supplementary Figures

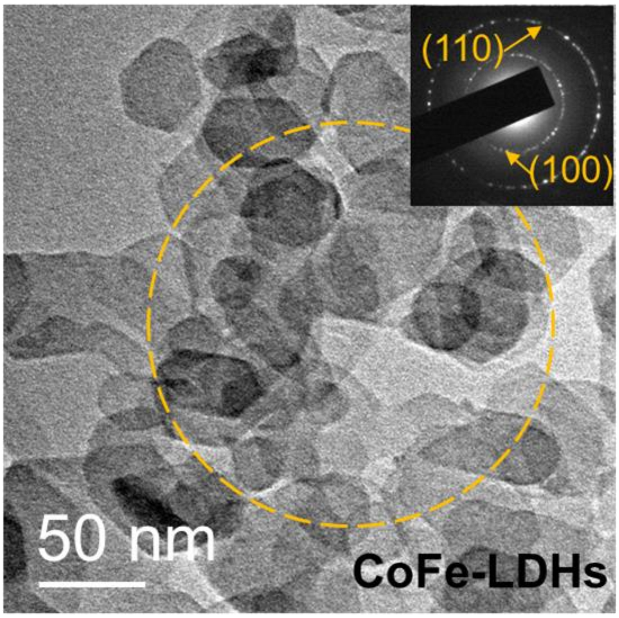

Supplementary Figure 1. The morphology of CoFe-LDHs. Transmission Electron Microscopy (TEM) images of as-prepared CoFe-LDHs, insets show corresponding Electron Diffraction (ED) pattern of a region of CoFe-LDHs nanosheets marked in yellow circle, showing LDHs crystalline structures.

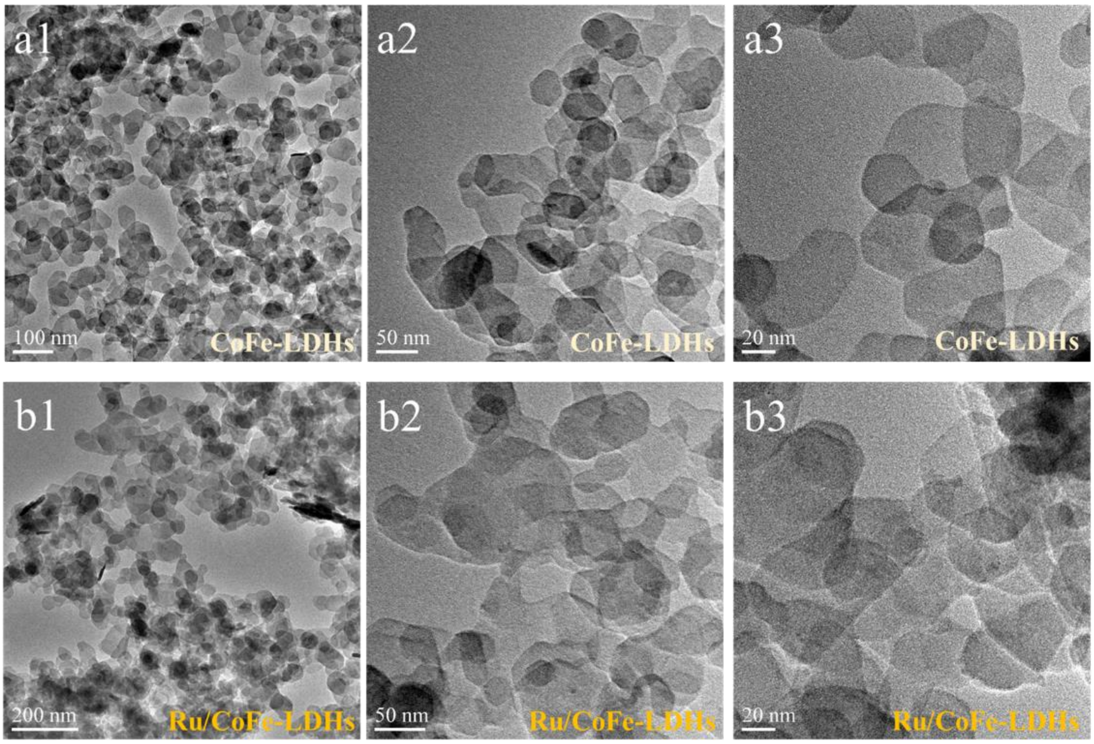

Supplementary Figure 2. The morphologies of CoFe-LDHs and Ru/CoFe-LDHs. Transmission Electron Microscopy (TEM) images of as-prepared CoFe-LDHs (a1, a2, and a3) and Ru/CoFe-LDHs (b1, b2, and b3) nanosheets with different ranges.

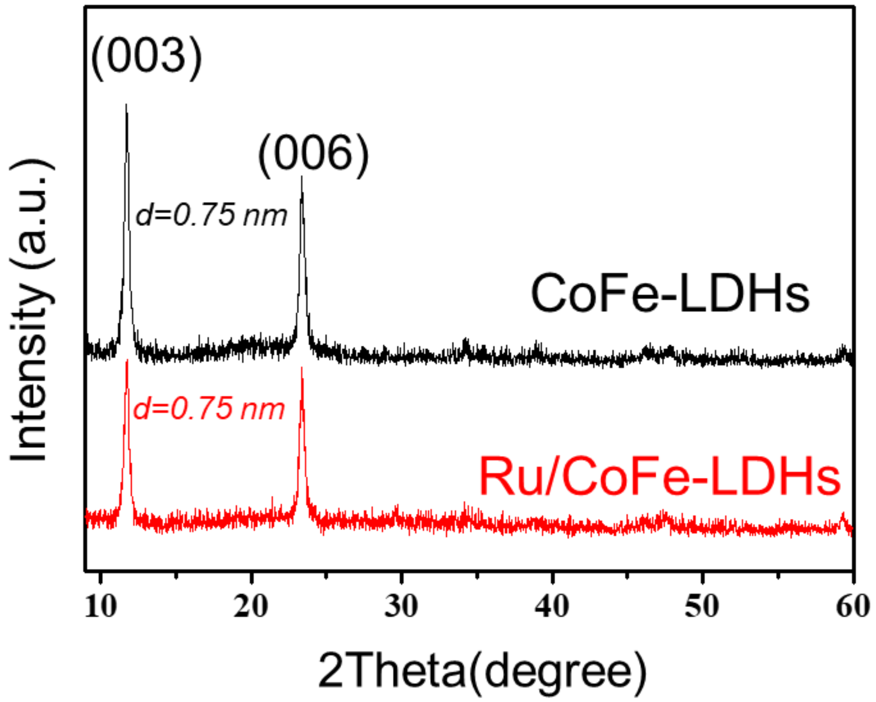

Supplementary Figure 3. The crystalline structures of CoFe-LDHs and Ru/CoFe-LDHs. XRD patterns of CoFe-LDHs and Ru/CoFe-LDHs.

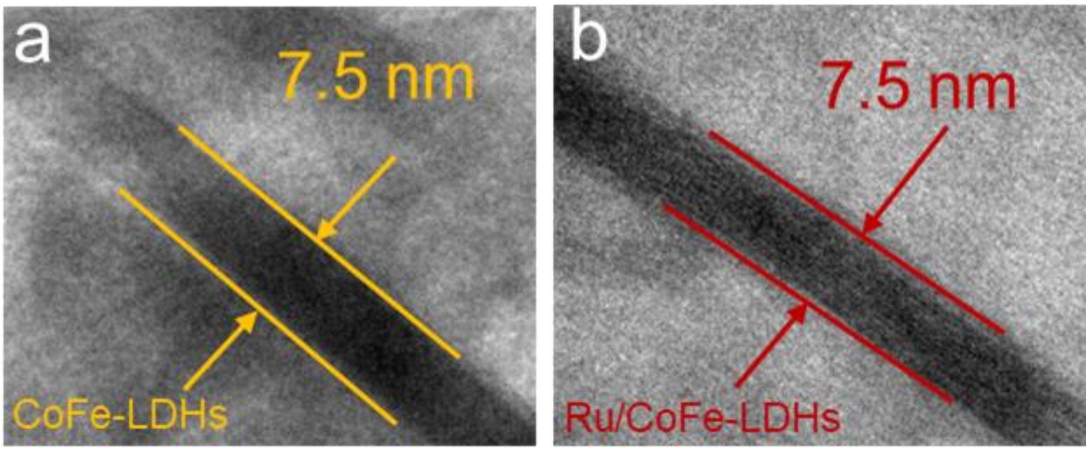

Supplementary Figure 4. The thickness of CoFe-LDHs and Ru/CoFe-LDHs. High-resolution transmission electron microscopy (HRTEM) images of CoFe-LDHs (a) and Ru/CoFe-LDHs (b).

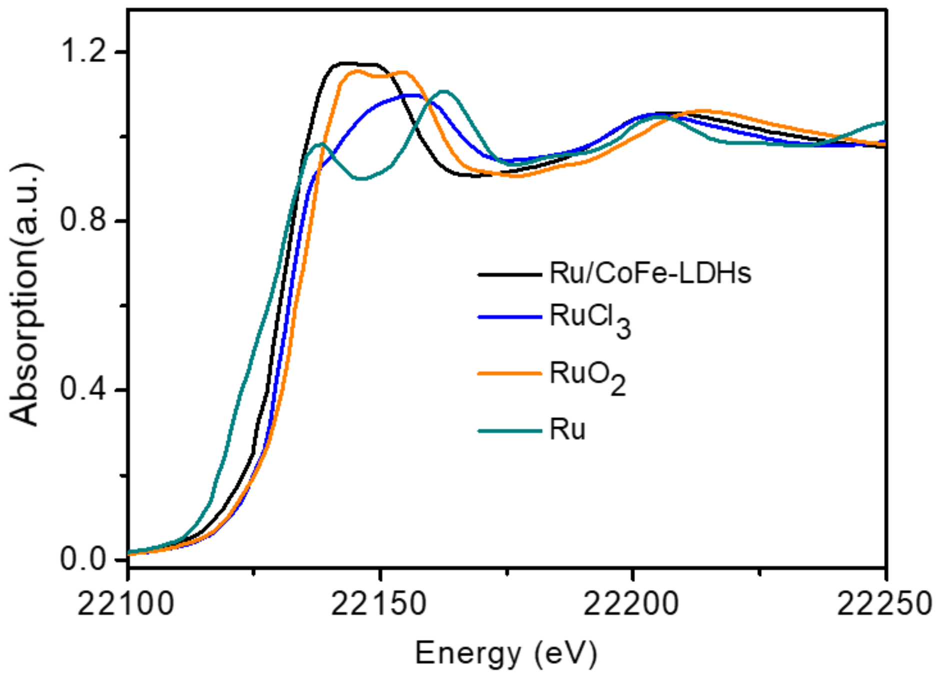

Supplementary Figure 5. XANES spectra at Ru *K*-edge. XANES spectra of Ru/CoFe-LDHs, $RuCl_3$, RuO_2 and Ru metal.

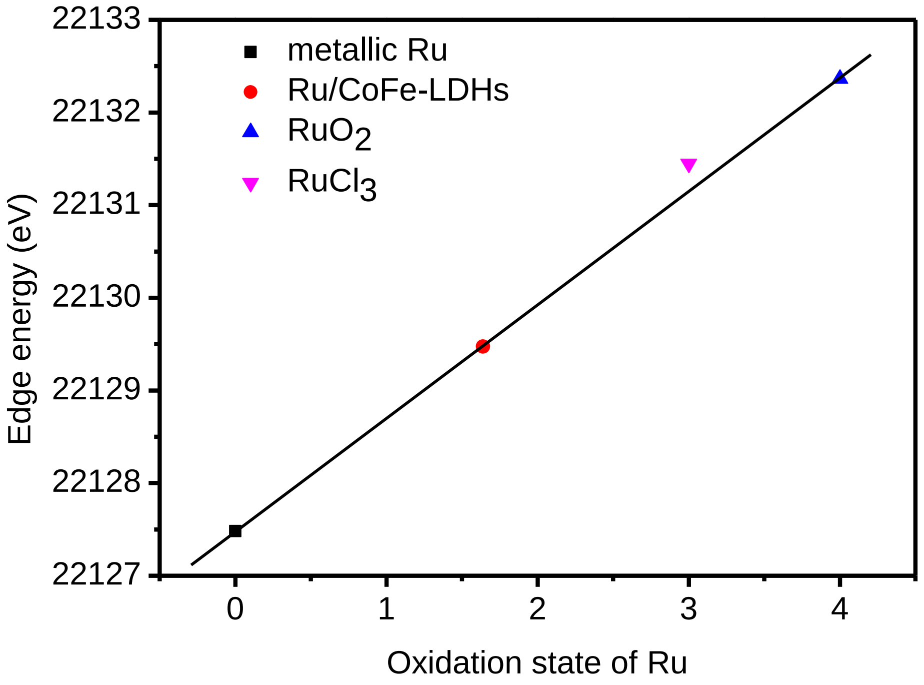

Supplementary Figure 6. Oxidation state analysis of Ru in different samples. Oxidation state fitting of Ru element in Ru/CoFe-LDHs by edge energy.

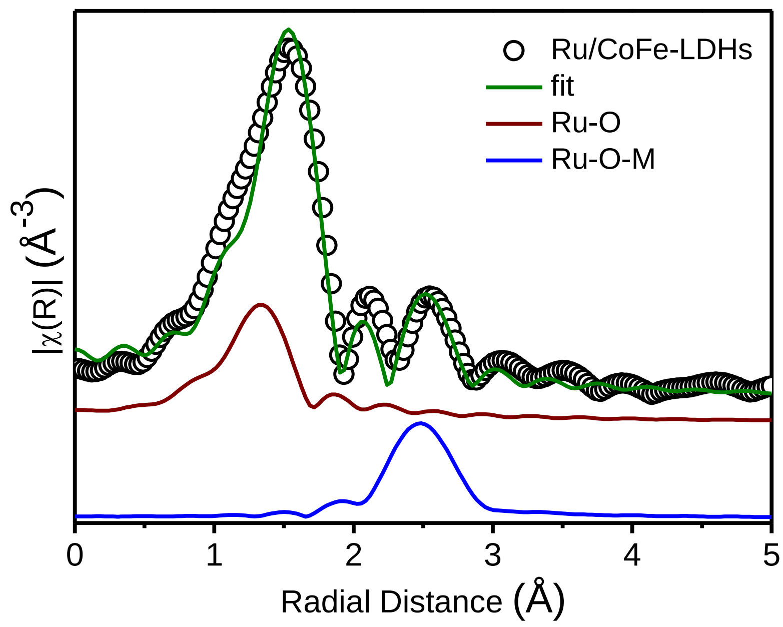

Supplementary Figure 7. FT-EXAFS spectra and fits of Ru k-edge. Model-based fits of Ru EXAFS for Ru/CoFe-LDHs and simulated EXAFS spectra from Ru-O and Ru-O-M (M=Co or Fe) bonds.

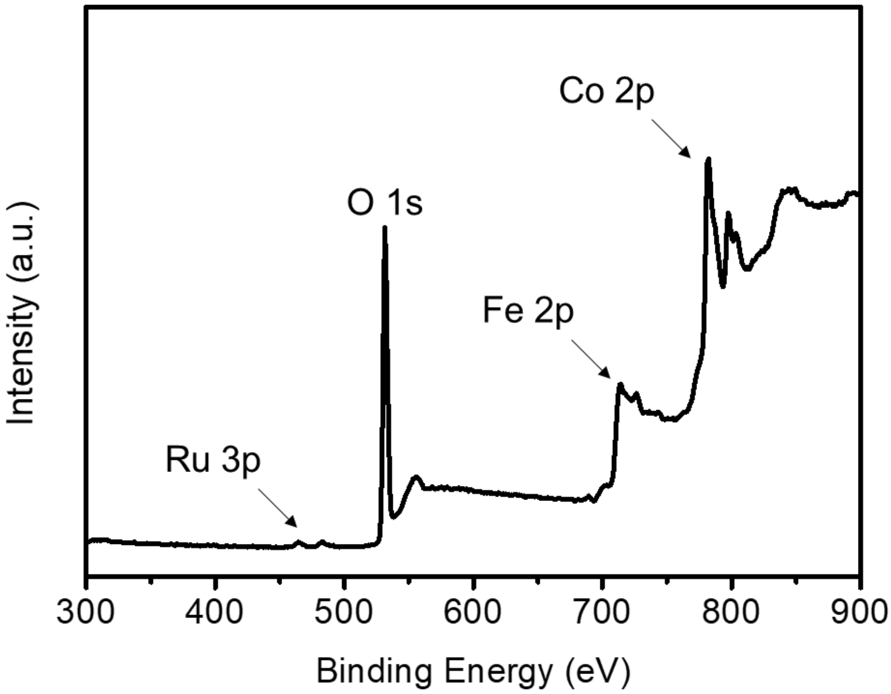

Supplementary Figure 8. The chemical compositions of Ru/CoFe-LDHs. X-ray photoelectron spectroscopy (XPS) survey spectra of Ru/CoFe-LDHs.

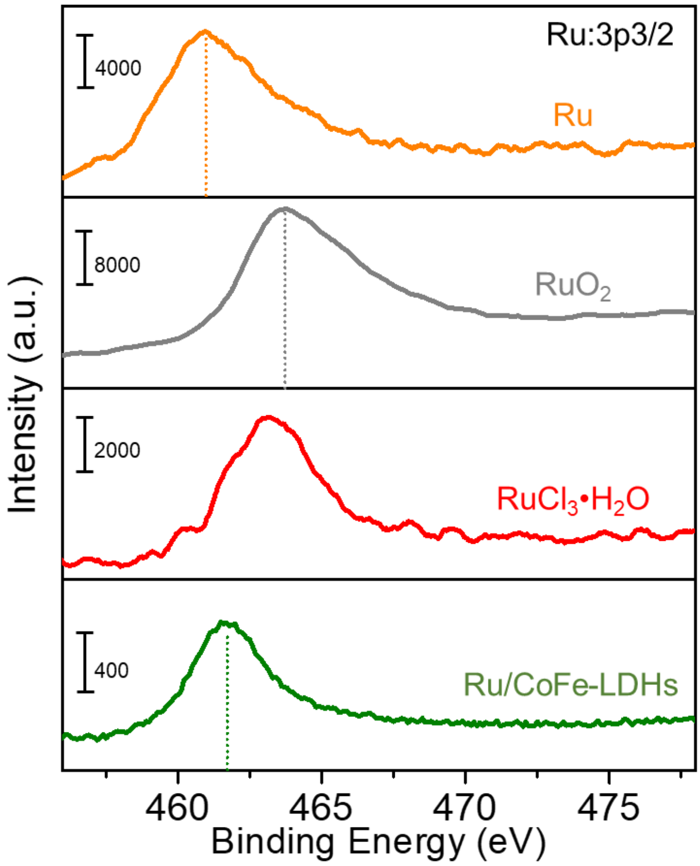

Supplementary Figure 9. Ruthenium oxidation states in our materials. High-resolution X-ray photoelectron spectroscopy (XPS) of Ru in metallic Ru, $RuCl_3$ hydrate, RuO_2 and Ru/CoFe-LDHs.

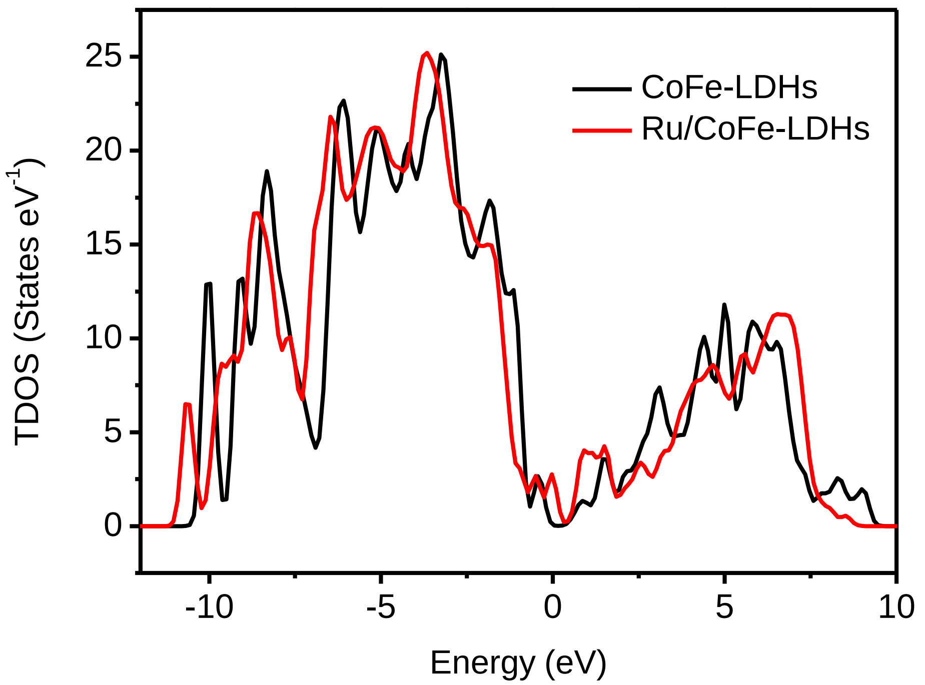

Supplementary Figure 10. The comparison of bandgap. Total density of states (TDOS) curves of Ru/CoFe-LDHs and CoFe-LDHs, the narrower bandgap of Ru/CoFe-LDHs indicates a more conductive structure.

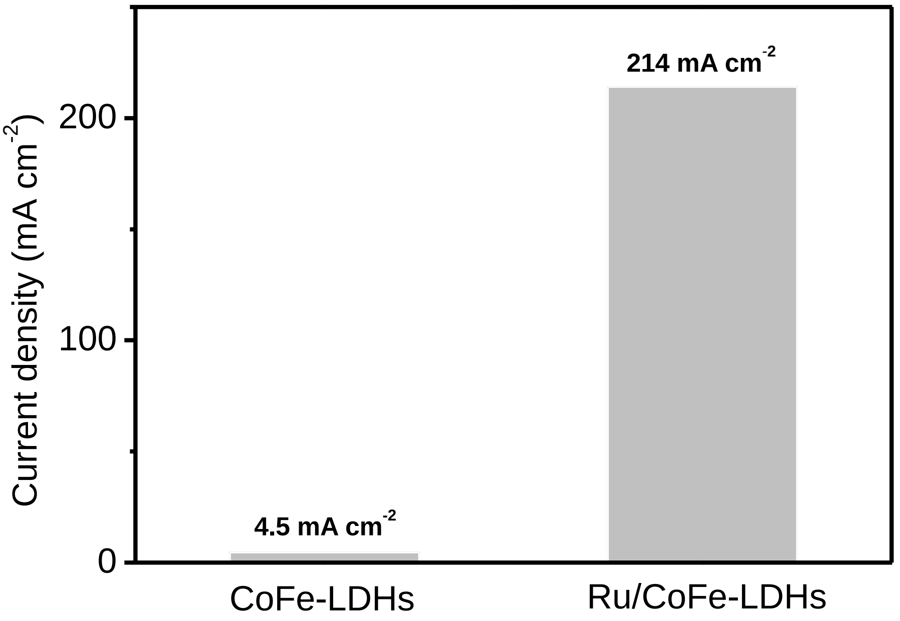

Supplementary Figure 11. The comparison of catalytic activity. Oxygen evolution reaction current density of as-prepared CoFe-LDHs and Ru/CoFe-LDHs catalysts at the potential of 1.5 V vs. RHE.

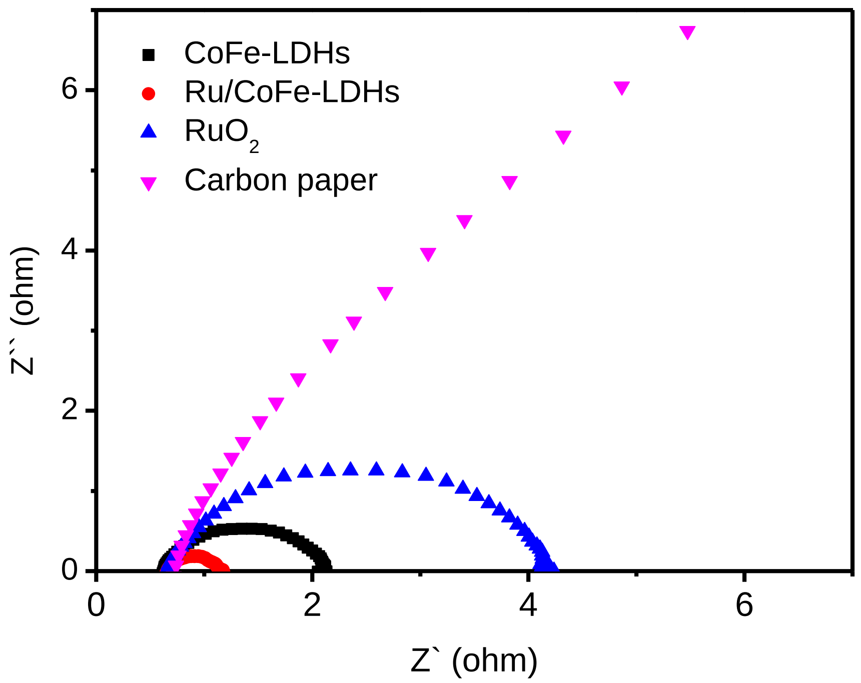

Supplementary Figure 12. The Comparison of impedance in OER. The EIS curves of Ru/CoFe-LDHs, CoFe-LDHs, RuO_2 and Carbon paper at the overpotential of 100 mV.

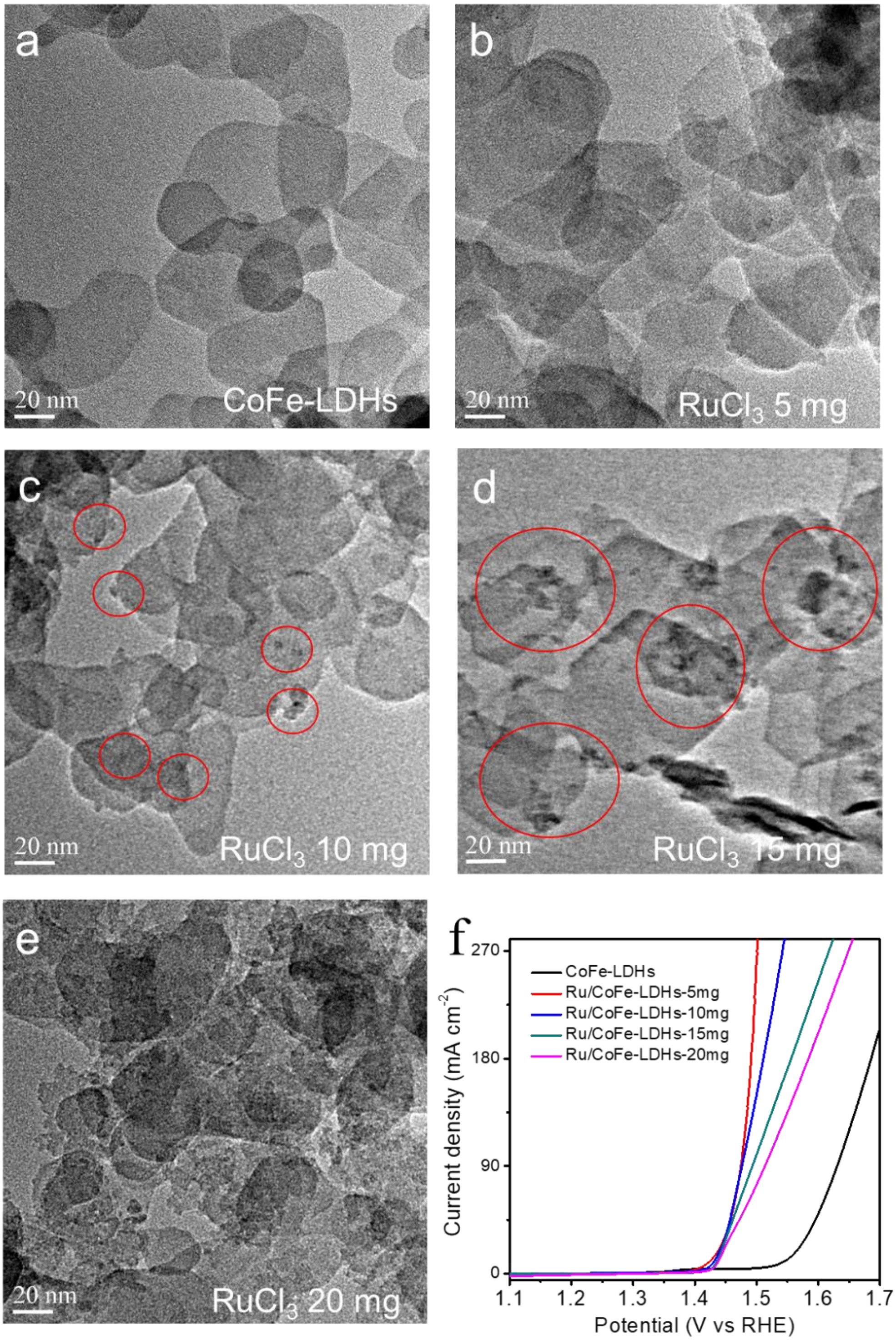

Supplementary Figure 13. The comparison of morphology and activity with the increase of Ru on the CoFe-LDHs surface. Transmission Electron Microscopy (TEM) images of as-prepared Ru/CoFe-LDHs with different raw material feeding amount of $RuCl_3$ hydrate, (a) 0mg, (b) 5mg, (c) 10mg, (d) 15mg, and (f) 20mg, respectively. (f) The corresponding polarization curves of the catalysts.

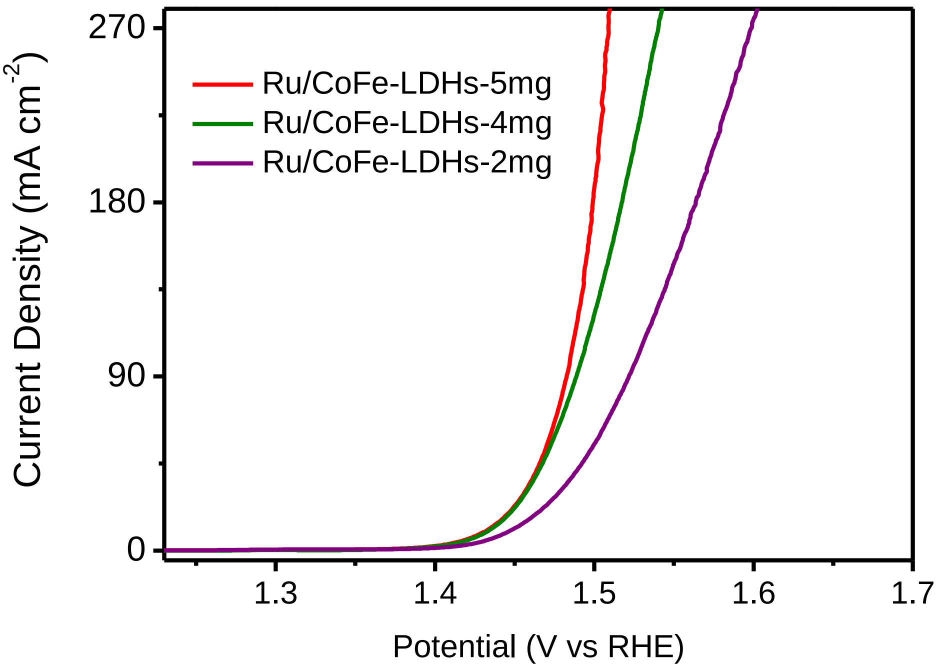

Supplementary Figure 14. The comparison of activity with the decrease of Ru on the CoFe-LDHs surface. The polarization curves of as-prepared Ru/CoFe-LDHs with different raw material feeding amount of $RuCl_3$ hydrate, 2mg, 4mg, and 5mg, respectively.

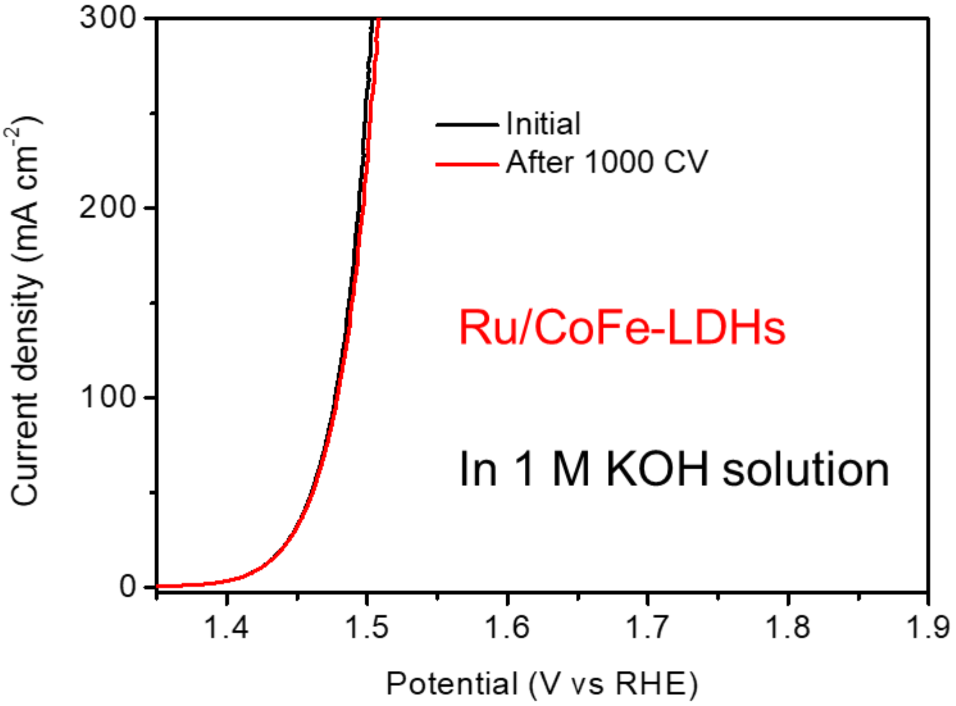

Supplementary Figure 15. Stability test for oxygen evolution reaction of the Ru/CoFe-LDHs catalyst. Negligible catalytic current density loss was observed after 1000 cycles.

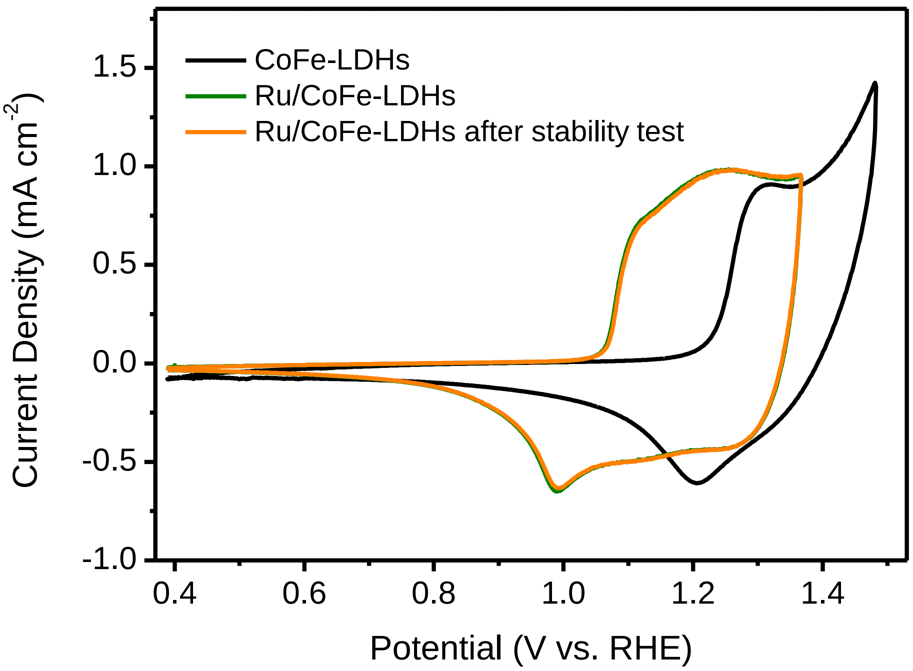

Supplementary Figure 16. The redox behaviors of the as-prepared catalysts. Cyclic voltammetry curves of CoFe-LDHs, Ru/CoFe-LDHs and the Ru/CoFe-LDHs after stability test, respectively. All the cyclic voltammetry curves were scanned in the fresh 1.0 M KOH electrolyte after 1-hour bubbling with argon gas.

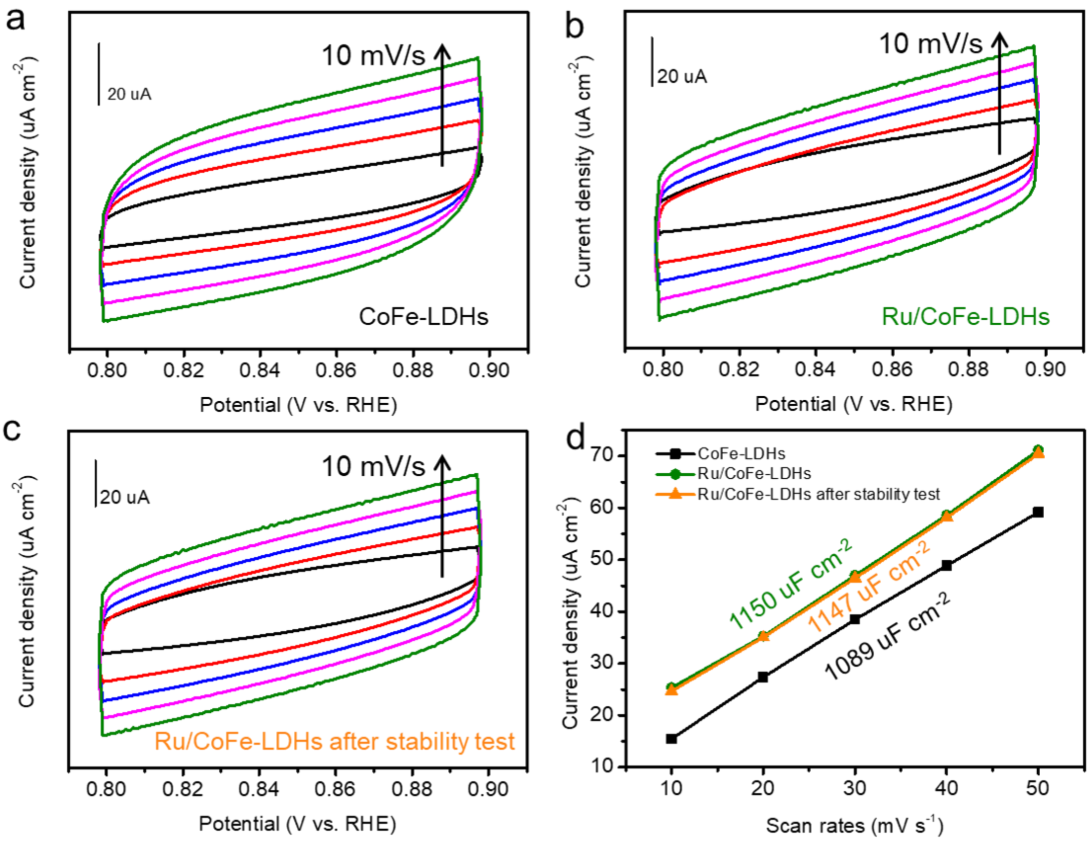

Supplementary Figure 17. Non-Faradic scan for double layer capacitance. (a,b,c) Electric double layer capacitance (C_{dl}) measurements at the non-Faradic region (0.8-0.9 V vs. RHE) with various scan rates (10 mV s^{-1}-50 mV s^{-1}) and (d) corresponding $2C_{dl}$ calculations of CoFe-LDHs, Ru/CoFe-LDHs and Ru/CoFe-LDHs after stability test, respectively. The slopes ($2C_{dl}$) were used to represent electrochemical active surface area (ECSA).

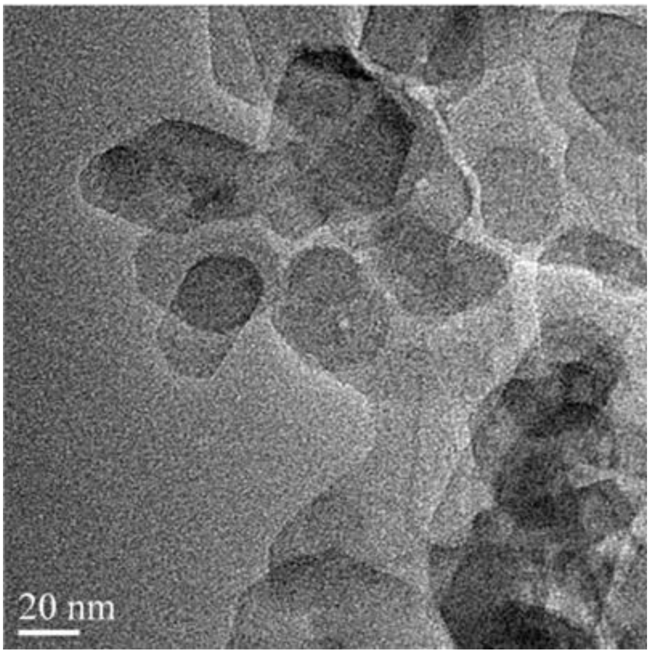

Supplementary Figure 18. The morphology of Ru/CoFe-LDHs after OER. TEM image of Ru/CoFe-LDHs after long term stability test.

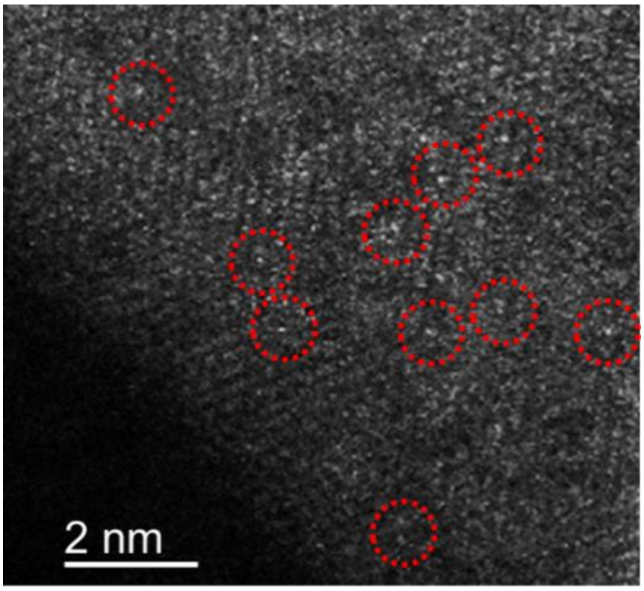

Supplementary Figure 19. The monatomic ruthenium on the surface of CoFe-LDHs after OER. Cs-corrected STEM image of Ru/CoFe-LDHs after stability test shows the monoatomic Ru dispersed on the surface of LDHs (some of the isolated Ru atoms are marked with red circles).

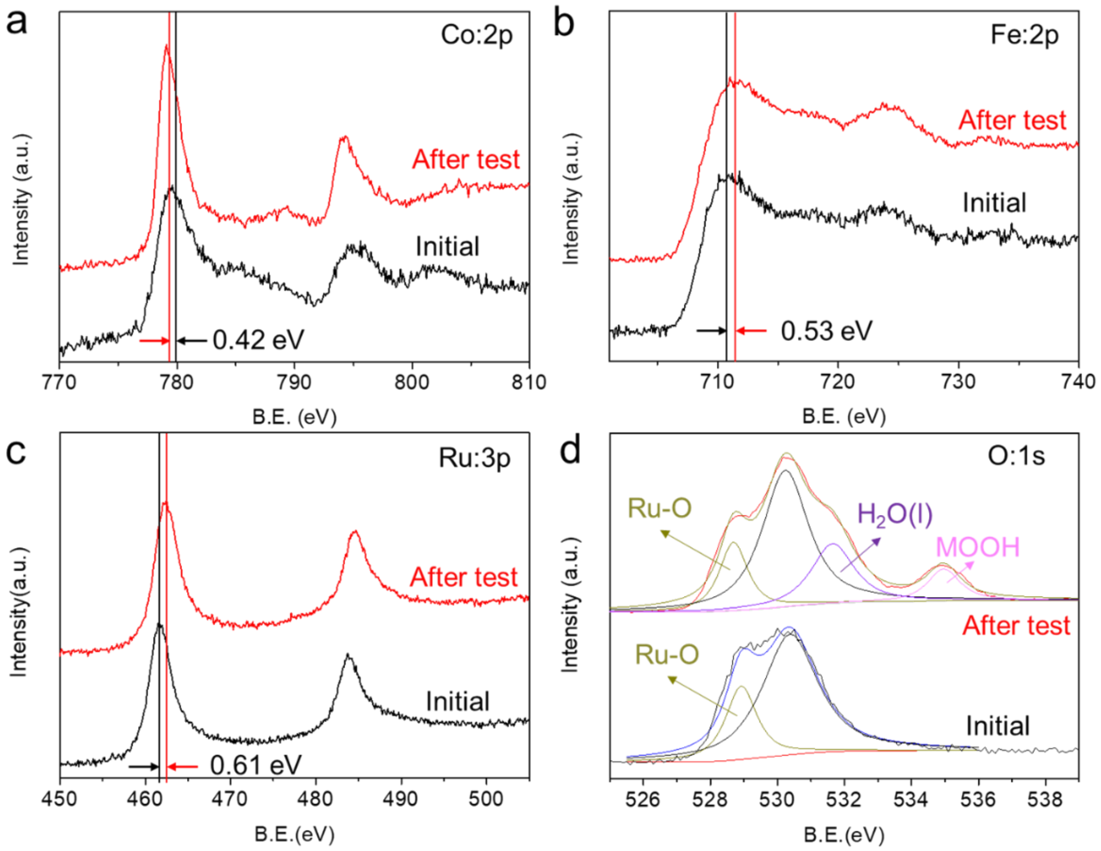

Supplementary Figure 20. The surface oxidation states of catalysts before and after OER. High-resolution X-ray photoelectron spectroscopy (XPS) of Co (a), Fe (b), Ru (c) and O (d) in the Ru/CoFe-LDHs before and after long term stability test.

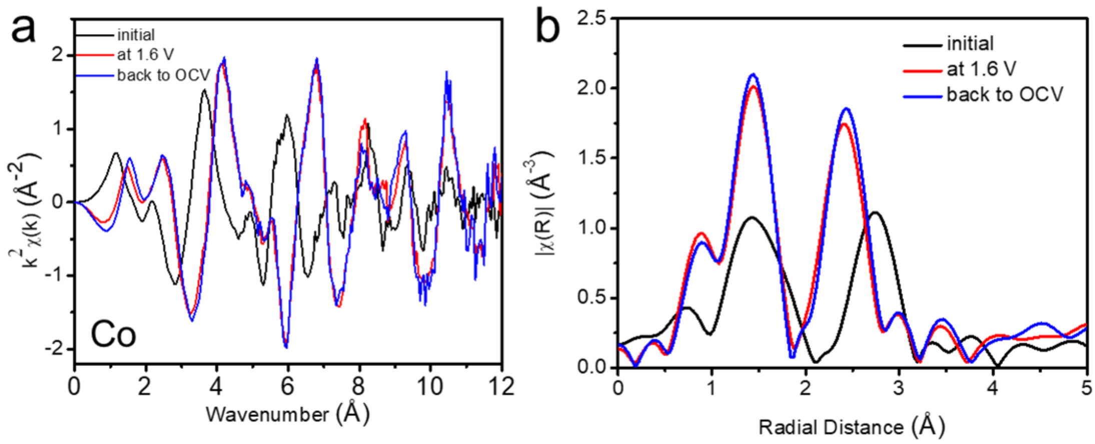

Supplementary Figure 21. Local structural change of Co in the OER process. Co *k*-space (a) and Fourier transfer R-space(b) of the *in-situ* extended X-ray absorption fine structure (EXAFS).

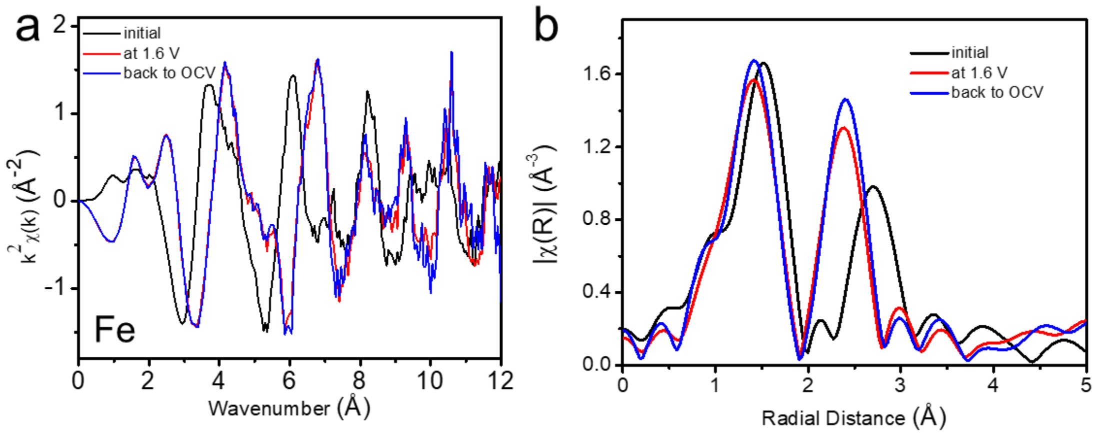

Supplementary Figure 22. Local structural change of Fe in the OER process. Fe *k*-space (a) and Fourier transfer R-space(b) of the *in-situ* extended X-ray absorption fine structure (EXAFS).

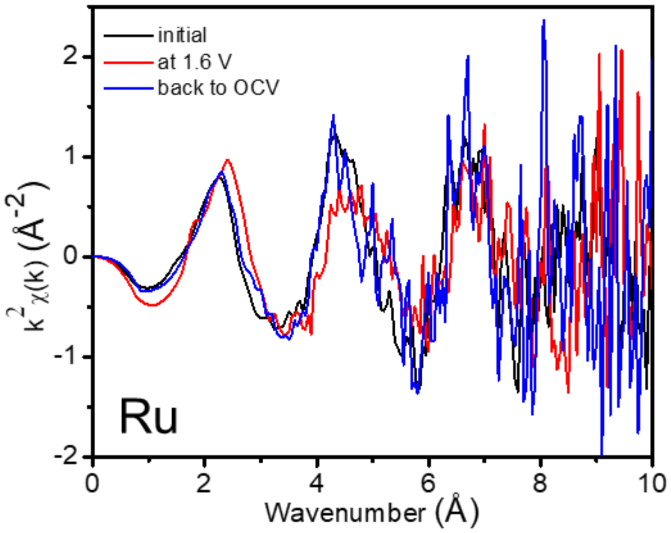

Supplementary Figure 23. Local structural change of Ru in the OER process. Ru *k*-space of the *in-situ* extended X-ray absorption fine structure (EXAFS).

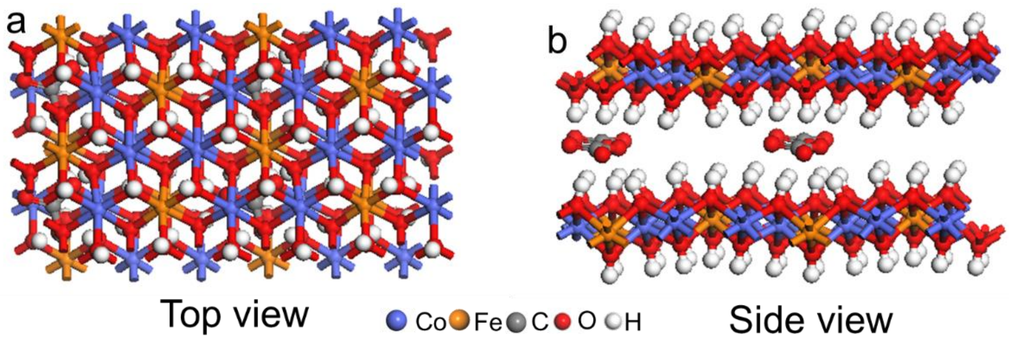

Supplementary Figure 24. Structural models of CoFe-LDHs. The schematic structures of CoFe-LDHs in top (a) and side (b) view.

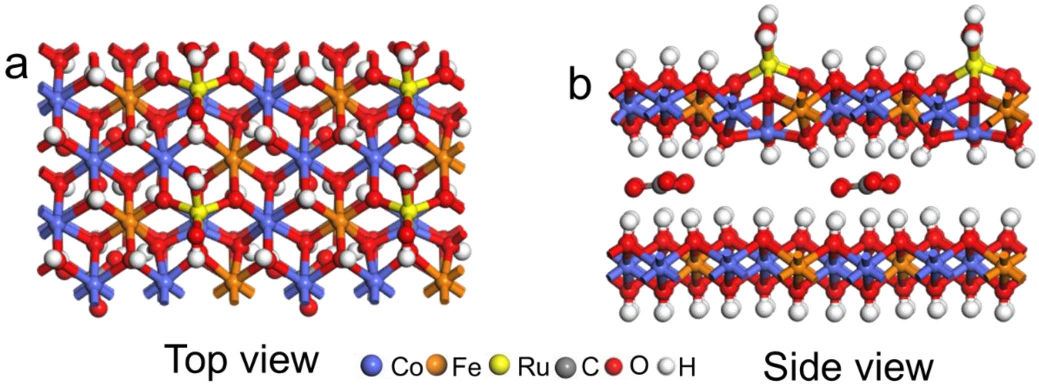

Supplementary Figure 25. Structural models of Ru/CoFe-LDHs.The schematic structures of Ru/CoFe-LDHs in top (a) and side (b) view and the Ru atom coordinating with five oxygen atoms.

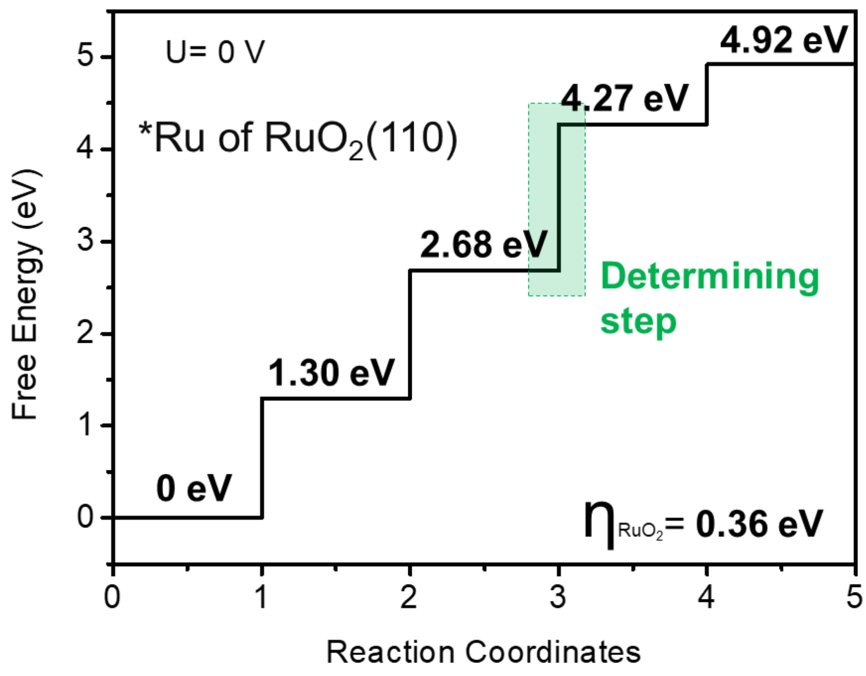

Supplementary Figure 26. Calculated free-energy diagram. Gibbs free-energy diagram for the four steps of OER on RuO_2.

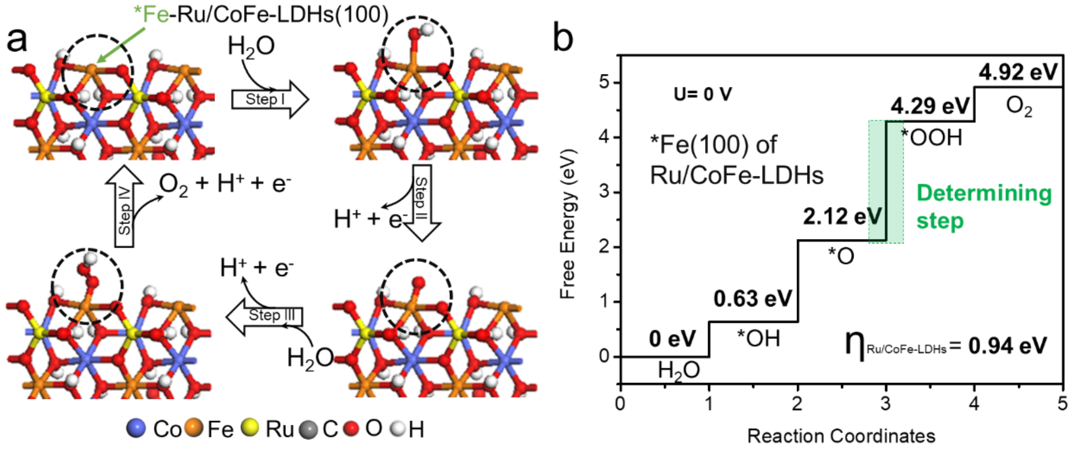

Supplementary Figure 27. Theoretical OER overpotential. Proposed 4e-mechanism of oxygen evolution reaction on Ru/CoFe-LDHs (a) for DFT+U calculation. The Fe ion (*) in the (100) crystal plane was the active site for Ru/CoFe-LDHs. Gibbs free-energy diagram for the four steps of OER on Ru/CoFe-LDHs (b). The green box step is the rate determining step and η stands for the overpotential.

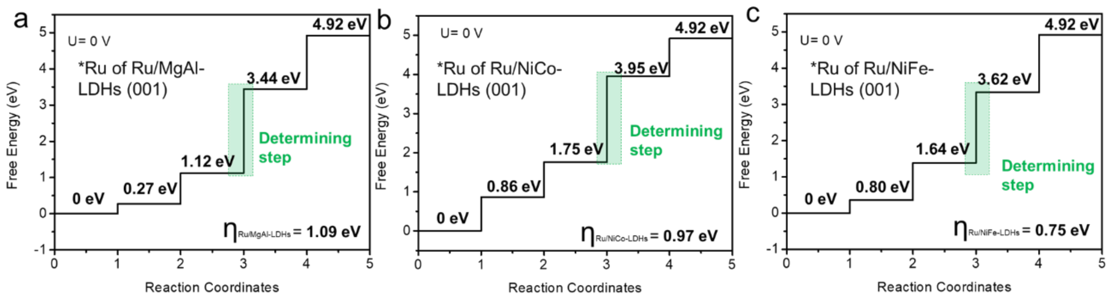

Supplementary Figure 28. Calculated free-energy diagrams. Ru ion (*) in the (001) crystal plane was the active site for samples. Gibbs free-energy diagram for the four steps of OER on Ru/MgAl-LDHs (a), Ru/NiCo-LDHs (b) and Ru/NiFe-LDHs (c). The green box step is the determining step and η stands for the overpotential.

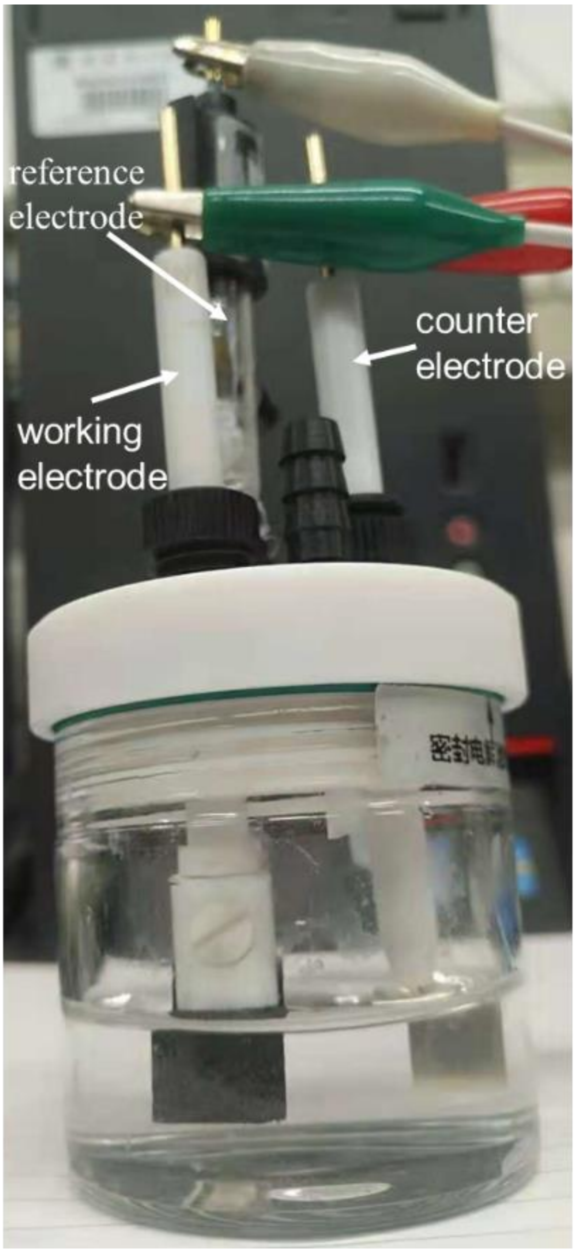

Supplementary Figure 29. The setup of the three-electrode glass cell

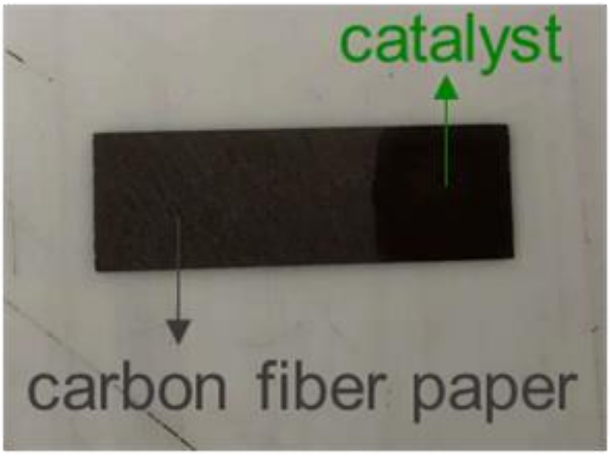

Supplementary Figure 30. The digital picture of the working electrode

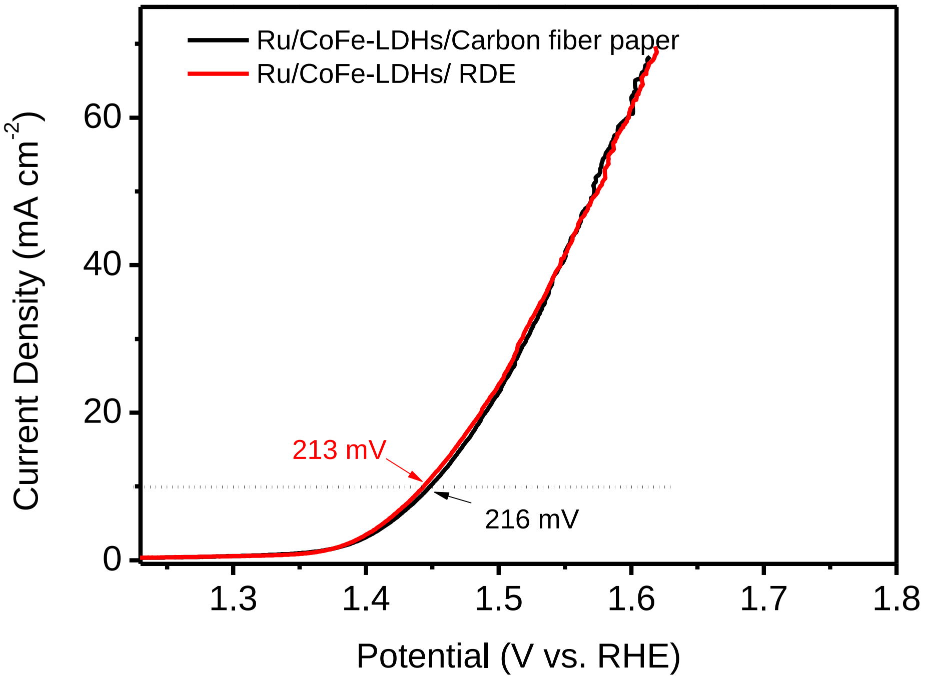

Supplementary Figure 31. The comparison of performance in different setups. The polarization curves of Ru/CoFe-LDHs in our setup and the rotating disk electrode (RDE) setup with the same mass loading (0.25 mg cm^{-2}). In these two setups, the catalyst exhibited a similar activity, which confirmed that there was no difference between our setup and the rotating disk electrode setup with 1.0 M KOH electrolyte. For our setup, the working electrode prepared by dropping of 10 uL catalyst ink onto the surface of carbon fiber paper (0.5 x 0.4 cm). For rotating disk electrode setup, we prepared the working electrode by dropping of 10 uL catalyst ink onto the surface of polished and cleaned glassy carbon rotating disc electrode (5 mm in diameter). During the linear sweep, rotating disk electrode was continuously rotated at 1600 rpm to remove the generated bubbles.

Supplementary Tables

Supplementary Table 1. Fitting parameters of the co-refined EXAFS spectra of Ru/CoFe-LDHs catalyst at different potentials (CN: coordination number; R: distance; E_0: energy shift; σ^2: mean-square disorder). The numbers in the parentheses are the last digital error.

**Ru initial**	CN	R (Å)	E_0 (eV)	σ^2 (Å^2)
Ru-O	3.9(7)	2.02(1)	2.17(1)	0.0026(2)
Ru-O-M(Co/Fe)	2.9(6)	3.05(3)	2.17(1)	0.0005(1)
**Co at 1.6V**	CN	R (Å)	E_0 (eV)	σ^2 (Å^2)
Co-O	2.0(4)	1.73(8)	2.23(3)	0.0168(9)
Co-O	4.0(8)	1.90(1)	2.23(3)	0.0006(9)
Co-O-Ru	2.0(4)	2.82(0.16)	2.23(3)	0.0200(9)
Co-O-Fe	2.0(4)	3.07(5)	2.23(3)	0.0014(7)
Co-O-Co	2.0(4)	2.84(0.17)	2.23(3)	0.0067(6)
**Co at OCV**	CN	R (Å)	E_0 (eV)	σ^2 (Å^2)
Co-O	3.4(4)	2.01(2)	2.39(9)	0.0033(5)
Co-O	1.7(2)	2.18(2)	2.39(9)	0.0007(5)
Co-O	1.7(2)	2.89(2)	2.39(9)	0.0008(5)
Co-O-Ru	1.7(2)	2.97(2)	2.39(9)	0.0315(3)
Co-O-Fe	1.7(2)	3.12(2)	2.39(9)	0.0030(6)
Co-O-Co	1.7(2)	3.13(2)	2.39(9)	0.0051(3)
**Fe at 1.6 V**	CN	R (Å)	E_0 (eV)	σ^2 (Å^2)
Fe-O	1.1(1)	1.81(1)	3.82(7)	0.0045(4)
Fe-O	1.1(1)	1.84(1)	3.82(7)	0.0029(9)
Fe-O	2.2(2)	1.93(2)	3.82(7)	0.0007(3)
Fe-O-Co	4.4(4)	2.82(1)	3.82(7)	0.0081(1)
Fe-O-Ru	1.1(1)	3.28(3)	3.82(7)	0.0051(1)
**Fe at OCV**	CN	R (Å)	E_0 (eV)	σ^2 (Å^2)
Fe-O	5.1(8)	2.00(1)	3.98(9)	0.0041(9)
Fe-O-Co	5.1(8)	3.10(2)	3.98(9)	0.0086(0)
Fe-O-Ru	0.9(4)	3.45(3)	3.98(9)	0.0046(1)

Supplementary Table 2. XPS quantitative analysis of Ru/CoFe-LDHs

	Atomic conc.%	Error %	Mass conc.%	Error %
Fe 2p	9.23	0.24	19.17	0.46
Co 2p	18.46	0.41	40.50	0.66
O 1s	51.71	0.34	30.77	0.33
C 1s	20.49	0.31	9.14	0.18
Ru 3p	0.11	0.01	0.42	0.04

Supplementary Table 3. Elemental analysis of Ru/CoFe-LDHs and electrolyte through ICP-MS measurements

Sample	Element	Test value
Ru/CoFe-LDHs	Ru	4.515±0.083 ppb[#]
Electrolyte of Ru/CoFe-LDHs (2 mg cm^{-2})	Ru	0.003±0.001 ppb[*]
	Fe	23.201±0.776 ppb
	Co	0.232±0.013 ppb
Electrolyte of RuO_2 (2 mg cm^{-2})	Ru	1951.163±1.854 ppb
Electrolyte of CoFe-LDHs (2 mg cm^{-2}) and RuO_2 (0.012 mg cm^{-2}) mixture	Ru	52.723±0.921 ppb
	Fe	20.448±0.675 ppb
	Co	0.203±0.016 ppb
Electrolyte of RuO_2 (0.012 mg cm^{-2})	Ru	86.374±0.815 ppb
Electrolyte of CoFe-LDHs (2 mg cm^{-2})	Fe	23.722±0.719 ppb
	Co	0.673±0.024 ppb

#″±″ means standard deviation (replicates=3).

*″0.003 ppb″ means the Ru in Ru/CoFe-LDHs is almost insoluble under OER working condition.

Supplementary Table 4. The electrocatalytic performance of as-prepared Ru/CoFe-LDHs comparing with some state-of-the-art catalytic electrodes.

Catalyst	Electrolyte	Mass loading (mg cm^{-2})	Current collector	Overpotential (mV)	Tafel slope (mV dec^{-1})	Reference
Ru/CoFe-LDHs	1.0 M KOH	1	Carbon fiber paper	198(10mA cm^{-2})*	39	*This work*
Ru/CoFe-LDHs	1.0 M KOH	0.25	Carbon fiber paper	216(10mA cm^{-2})	N	*This work*
Ru/CoFe-LDHs	1.0 M KOH	0.25	GCE$^{\&}$	213(10mA cm^{-2})	N	*This work*
NiFe-MOF array	0.1 M KOH	0.3	GCE	240(10mA cm^{-2})	34	1
Co-B_i NS/graphene	1.0 M KOH	0.29	GCE	290(10mA cm^{-2})	53	2
CoMnP	1.0 M KOH	0.284	GCE	330(10mA cm^{-2})	61	3
Co-C_3N_4/CNT	0.1 M KOH	0.4	GCE	380(10mA cm^{-2})	68.4	4
Co_3Ni_1P	1.0 M KOH	0.64	GCE	281(10mA cm^{-2})	66.5	5
E-CoFe LDH	1.0 M KOH	0.204	GCE	300(10mA cm^{-2})	41	6
RuO_2	1.0 M KOH	0.1	GCE	370(10mA cm^{-2})	105	7
Co@CoO/NG	1.0 M KOH	2	Carbon fiber paper	315(10mA cm^{-2})	68	8
CoO/CNF	0.1 M KOH	0.6	Carbon fiber paper	360(10mA cm^{-2})	69.8	9
Co_1Mn_1CH/NF	1.0 M KOH	N	Nickel foam	294(30mA cm^{-2})	N	10
NiFe LDH/NF	1.0 M KOH	N	Nickel foam	240(10mA cm^{-2})	N	11
FeNi-rGO LDH	1.0 M KOH	0.25	Nickel foam	195(10mA cm^{-2})	39	12
CoN/NF	1.0 M KOH	1.5	Nickel foam	290(10mA cm^{-2})	70	13
Co_3O_4	1.0 M KOH	N	Nickel foam	290(10mA cm^{-2})	84	14
$Ni_xFe_{1-x}Se_2$-DO	1.0 M KOH	N	Nickel foam	195(10mA cm^{-2})	28	15
IrO_2 /NF	1.0 M KOH	0.7	Nickel foam	285 (10mA cm^{-2})	46	16

$^{\&}$"GCE" means glassy carbon electrode.

* "198 (10 mA cm^{-2})" means that the OER operating voltage of the corresponding catalyst is 198mV for obtaining j_{OER}=10 mA cm^{-2}.

Supplementary Table 5. XPS quantitative analysis of Ru/CoFe-LDHs after stability test

	Atomic conc.%	Error %	Mass conc.%	Error %
Fe 2p	8.93	0.31	18.81	1.47
Co 2p	17.86	0.42	39.74	0.93
O 1s	52.87	0.47	31.91	0.83
C 1s	20.24	0.49	9.14	0.32
Ru 3p	0.10	0.02	0.40	0.03

Supplementary References

1 Duan, J., Chen, S. & Zhao, C. Ultrathin metal-organic framework array for efficient electrocatalytic water splitting. *Nature Commun.* **8**, 15341 (2017).

2 Chen, P. *et al.* Strong-Coupled Cobalt Borate Nanosheets/Graphene Hybrid as Electrocatalyst for Water Oxidation Under Both Alkaline and Neutral Conditions. *Angew. Chem. Int. Edit.* **55**, 2488-2492 (2016).

3 Li, D., Baydoun, H., Verani, C. N. & Brock, S. L. Efficient Water Oxidation Using CoMnP Nanoparticles. *J. Am. Chem. Soc.* **138**, 4006-4009 (2016).

4 Zheng, Y. *et al.* Molecule-Level g-C_3N_4 Coordinated Transition Metals as a New Class of Electrocatalysts for Oxygen Electrode Reactions. *J. Am. Chem. Soc.* **139**, 3336-3339 (2017).

5 Fu, S. *et al.* Highly Ordered Mesoporous Bimetallic Phosphides as Efficient Oxygen Evolution Electrocatalysts. *ACS Energy Lett.* **1**, 792-796 (2016).

6 Zhou, P. *et al.* Acid-etched layered double hydroxides with rich defects for enhancing the oxygen evolution reaction. *Chem. Commun.* **53**, 11778-11781 (2017).

7 Fu, S. *et al.* Ultrafine and highly disordered Ni_2Fe_1 nanofoams enabled highly efficient oxygen evolution reaction in alkaline electrolyte. *Nano Energy* **44**, 319-326 (2018).

8 Zhang, S. *et al.* N-Doped graphene-supported Co@CoO core–shell nanoparticles as high-performance bifunctional electrocatalysts for overall water splitting. *J. Mater. Chem. A* **4**, 12046-12053 (2016).

9 Wang, H. *et al.* Bifunctional non-noble metal oxide nanoparticle electrocatalysts through lithium-induced conversion for overall water splitting. *Nature Commun.* **6**, 7261 (2015).

10 Tang, T. *et al.* Electronic and Morphological Dual Modulation of Cobalt Carbonate Hydroxides by Mn Doping toward Highly Efficient and Stable Bifunctional Electrocatalysts for Overall Water Splitting. *J. Am. Chem.Soc.* **139**, 8320-8328 (2017).

11 Luo, J. *et al.* Water photolysis at 12.3% efficiency via perovskite photovoltaics and Earth-abundant catalysts. *Science* **345**, 1593 (2014).

12 Long, X. *et al.* A Strongly Coupled Graphene and FeNi Double Hydroxide Hybrid as an Excellent Electrocatalyst for the Oxygen Evolution Reaction. *Angew. Chem. Int. Edit.* **126**, 7714-7718 (2014).

13 Zhang, Y. *et al.* Rapid Synthesis of Cobalt Nitride Nanowires: Highly Efficient and Low-Cost Catalysts for Oxygen Evolution. *Angew. Chem. Int. Edit.* **55**, 8670-8674 (2016).

14 Zhu, Y. P., Ma, T. Y., Jaroniec, M. & Qiao, S. Z. Self-Templating Synthesis of Hollow Co_3O_4 Microtube Arrays for Highly Efficient Water Electrolysis. *Angew. Chem. Int. Edit.* **56**, 1324-1328 (2017).

15 Xu, X., Song, F. & Hu, X. A nickel iron diselenide-derived efficient oxygen-evolution catalyst. *Nature Commun.* **7**, 12324 (2016).

16 Yan, X. *et al.* From Water Oxidation to Reduction: Transformation from $Ni_xCo_{3-x}O_4$ Nanowires to NiCo/$NiCoO_x$ Heterostructures. *ACS Appl. Mater. Inter.* **8**, 3208-3214 (2016).
